# Effects of a Mouthrinse Containing Silver Nanoparticles on Polymicrobial Oral Biofilms

**DOI:** 10.3290/j.ohpd.b5816545

**Published:** 2024-11-07

**Authors:** Kiyoshi Tomiyama, Kiyoko Watanabe, Junko Iizuka, Nobushiro Hamada, Yoshiharu Mukai

**Affiliations:** a Associate Professor, Department of Restorative Dentistry, Faculty of Dentistry, Yokosuka, Japan. Study concept and design, wrote the manuscript, collected, assembled, analysed, and interpreted data, approved the manuscript for publication.; b Associate Professor, Department of Liberal Arts Education, Faculty of Dentistry, Yokosuka, Japan. Data analysis and interpretation, approved the manuscript for publication.; c Associate Professor, Department of Restorative Dentistry, Faculty of Dentistry, Yokosuka, Japan. Collection and assembly of data, approved the manuscript for publication.; d Professor, Department of Oral Microbiology, Faculty of Dentistry, Yokosuka, Japan. Administrative support, approved the manuscript for publication.; e Professor, Department of Restorative Dentistry, Faculty of Dentistry, Yokosuka, Japan. Administrative support, approved the manuscript for publication.

**Keywords:** chlorhexidine, dental biofilm, preventive dentistry, silver nanoparticles.

## Abstract

**Purpose:**

To investigate the antimicrobial effects of a mouthrinse containing silver nanoparticles (AgNP) on polymicrobial biofilms in vitro.

**Materials and Methods:**

Polymicrobial biofilms were grown on glass cover slips following the method of Exterkate. Saliva collected from a healthy human was added to McBain medium (including 0.2% sucrose) to achieve a 50-fold dilution. Glass coverslips were attached to the lid of a 24-well culture plate and suspended in the medium of each well. After 24 h of cultivating, coverslips with biofilms were immersed in each of four treatment solutions or sterile deionized water for 5 min. The control and four treatment groups were as follows: 1) control: sterile deionized water; 2) nanosilver (NS): mouthrinse containing AgNP; 3) 0.05C: 0.05% chlorhexidine gluconate; 4) 0.2C: 0.2% chlorhexidine gluconate; 5) Xyl: 25% xylitol. The biofilms were further regrown for 48 h. After removing the biofilms ultrasonically, they were cultured on blood agar, viable cells were counted, and the amount of lactic acid in the biofilms was analysed using a colorimetric assay.

**Results:**

Mouthrinse containing AgNP suppressed viable cells in the biofilm to the same degree or more than with chlorhexidine gluconate. Amounts of lactic acid after 72 h cultivation of biofilms treated with 0.2C and NS showed consistently low values.

**Conclusion:**

The mouthrinse containing AgNP suppressed viable cells in polymicrobial biofilms to the same level as 0.2% chlorhexidine or higher.

Most research on bacterial pathogenesis has focused on acute infections. Less is known about the pathogenesis of infections caused by bacteria that grow as aggregates in biofilms. Such infections tend to be chronic, resisting innate and adaptive immune defense mechanisms and antibiotics, and their treatment presents a considerable unmet clinical need.^2^ To date, several approaches are in early-stage development, but there are as yet no drugs specifically targeting bacteria in biofilms.

Nanoparticles (NP) have broad applications owing to their small size and high surface-to-volume ratio, and are currently being extensively studied for their antimicrobial and anti-biofilm activities.^16^ NP are a promising therapeutic approach due to their capacity to deliver drugs to the target site in the optimum dosage range, protect them against deactivation, and increase their therapeutic efficiency with fewer side effects.^15^ The nano-formulations have high selectivity for bacterial cells and can cross biological barriers such as biofilm due to their small size, large surface area, and highly reactive nature.^3^ The small size of NP enables them to penetrate biofilms and microbial cell walls, while their high surface area facilitates drug loading.^17^ Also, due to their property of facile cellular uptake, AgNP can be delivered close to bacteria within biofilms, where conventional antibacterial agents often struggle to penetrate, as demonstrated in a recent study.^8^ Additionally, NP exhibit long plasma half-lives and are easily excreted through the kidneys.^3^ The oral administration of AgNPs showed that the nanoparticles were less absorbed, reaching a higher fecal excretion and lower levels in organs.^23^ Indeed, when citrate-coated nanoparticles were orally administered to rats, it was found that AgNP blood levels were very low, and high amounts of nanoparticles were found in the feces.^13^

Silver nanoparticles (AgNP) represent a major group of NP with extraordinary potential in combating multidrug-resistant bacterial pathogens, offering an alternative approach to treating bacterial infections. However, the role of AgNP as efficient biofilm inhibitors and their effects on extracellular polymeric substances have not been fully elucidated. AgNP are known to be effective against disease-causing pathogens, including the influenza virus^7^ and norovirus.^14^ Additionally, AgNP exhibit effectiveness against single-celled bacteria and fungi while also inhibiting mold growth. Their strong bactericidal activity suppresses the proliferation of odor-causing bacteria, and they exhibit an excellent deodorizing effect by absorbing compounds such as formaldehyde and ammonia.^10^

A polymicrobial (PM) biofilm model^5^ reproduces the oral bacterial flora outside the oral cavity and is useful for rapid analysis of the effects of antibacterial agents and materials. In this study, we investigated the effect of a mouthrinse containing AgNP on PM biofilms formed outside the mouth using an ex-vivo biofilm model derived from human saliva.

The purpose of this study was to analyse the persistence of the antibacterial effect of AgNP on PM biofilms formed by multispecies oral bacteria within the oral cavity.

## MATERIALS AND METHODS

### Experimental Groups

The control and treatment groups consisted of the following: (1) control: sterile deionized water; (2) 0.05C: 0.05% solution chlorhexidine gluconate (CHX); (3) 0.2C: 0.2% CHX (Corsodyl, Glaxo SmithKline; Brentford, UK); (4) Xyl: 25 vol% xylitol (Fujifilm Wako Pure Chemical; Osaka, Japan); (5) nanosilver (NS) mouthrinse (Elementa; Payson, UT, USA), a commercially available product containing AgNP (Table 2). The NS mouthrinse used in this study contained 25% xylitol. To clarify the potential antibacterial effects of this ingredient and NS, we included 25% xylitol as a treatment group.

Table 1 displays the components of NS. The 0.05C solution was prepared by diluting the 0.2% CHX solution with sterilised distilled water. Each group consisted of 12 samples.

**Table 1:** Components of the mouthrinse containing silver nanoparticles (AgNPs)

Component	wt %
Deionized water	70.45%
Xylitol	25.0%
Calcium acetate	0.90%
Nano silver	0.475%
Wintergreen flavor	3.17%


### Specimens

Glass coverslips (diameter 12 mm, thickness 0.15 mm, Menzel; Braunschweig, Germany) served as substrates for growing PM biofilms. A total of 120 glass coverslips (experimental size 12×5 groups × 2 biofilm growth periods) were used for counting colony-forming units (CFU) and analysing the amounts of lactic acid production. After assembling the lid and specimens, they were autoclaved.

### Saliva Collection

The study protocol was approved by the Research Ethics Committee of Kanagawa Dental University (Approval number: 445).

Saliva collection adhered to the ethical standards of the committee responsible for human experimentation. Saliva was collected from one healthy adult donor with natural dentition and no active caries or acute periodontal disease. The donor refrained from taking antibiotics, using oral rinse, and brushing teeth for 24 h, as well as ingesting food or drink for 2 h before saliva collection. Stimulated saliva was obtained by chewing Parafilm M Barrier Film (Pechiney Plastic Packaging; Chicago, IL, USA) and kept on ice. Following filtration with sterile glass wool, the saliva was diluted to a 70% concentration in sterile glycerol and stored at –80°C.

### Preparation of PM Biofilms (Fig 1)

**Fig 1:**
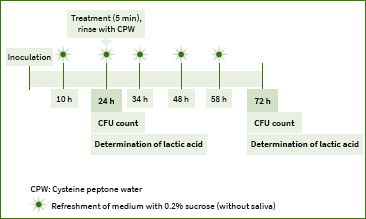
Experimental schedule for the biofilm growth, treatment and analysis.

PM biofilm preparation followed a method described by Exterkate et al.^5^ The biofilms were formed on glass coverslips using the diluted saliva in a high-throughput active attachment model. The inoculation medium for PM biofilms was 50-fold diluted saliva in a semi-defined medium comprising 2.5 g/l mucin, 2.0 g/l Bacto Peptone, 2.0 g/l trypticase peptone, 1.0 g/l yeast extract, 0.35 g/l NaCl, 0.2 g/l KCl, 0.2 g/l CaCl_2_, 0.001 g/l hemin, and 0.0002 g/l vitamin K1 with 0.2% sucrose and 50 mmol/l PIPES at pH 7.0. Biofilms were grown on glass coverslips in a buffered McBain medium.

After preparing the medium, biofilms were produced by adding 1.5 ml of the inoculation medium to each well of standard polystyrene 24-well plates (Greiner Bio-One Japan; Tokyo, Japan) followed by anaerobic incubation (10% CO_2_, 10% H_2_, and 80% N_2_) for 10 h at 37°C.

The lid was then transferred to a new plate containing fresh medium (without saliva) and incubated for a further 14 h. After 24 h of culture, the lid with specimens was transferred to a new plate containing 1.5 ml/well of antimicrobial agents or sterilised deionized water, followed by incubation for 5 min at room temperature.

Subsequently, the lid was transferred to a new plate containing 2 ml of CPW (cysteine peptone water) and moved up and down in the solution 10 times to stop the effects of antimicrobial agents. This procedure was repeated three times with fresh CPW.

The specimens were then anaerobically cultured for up to 72 h by replacing the culture medium every 10 and 14 h to form a biofilm (CO_2_: 10%; H_2_: 10%; N_2_: 80%; 37°C). Finally, specimens were harvested using ultrasonic waves (Transsonic T780, Elma electric; Stuttgart, Germany), followed by vortexing for 30 s (Tube mixer, VTX-3500, LMS; Tokyo, Japan).

### Determination of CFU Counts

After treatment, coverslips with biofilms were immediately transferred into tubes containing 2 ml of CPW. Subsequently, the biofilms were harvested by ultrasonication (Transsonic T780; Elma Electric), followed by vortexing for 30 s (VTX-3500 Tube Mixer; LMS). The harvested biofilms were then serially diluted in CPW, and the PM bacteria were plated on tryptic soy agar blood plates. The plates were incubated for 96 h at 37°C under anaerobic conditions (10% CO_2_, 10% H_2_, and 80% N_2_) and the total number of colonies (CFU/ml) was counted.

### Acid Production Assay

At the end of the biofilm formation period, and when applicable, after treatment, the lid was placed on a new plate containing 1.5 ml/well of buffered peptone water with 0.2% sucrose. The model was then incubated anaerobically for 3 h at 37°C. Following incubation, the amount of lactic acid (mmol/l) formed during this period was analysed using a colorimetric assay.^22^

### Statistical Analysis

The total bacterial count (CFU/ml) and lactate production (mmol/l) were analysed at the time of each 5 min treatment on the biofilm, immediately after the treatments, and after an additional 48 h of culture. The measured values were statistically analysed using one-way ANOVA and Tukey’s test with p < 5% defined as statistically significant, using SPSS-PC software version 28.0.1 (SPSS; Tokyo, Japan).

## RESULTS

### Analysis of Viable Cells

**Fig 2:**
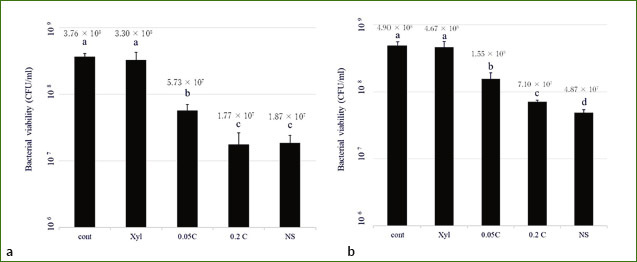
Bacterial viability in biofilms after treatment (a), and following 48 h cultivation after treatment (b). Values with different letters differ statistically significantly among groups (p < 0.05).

Table 2 shows the abbreviations of the solutions used. The number of bacterial cells (CFU/ml) after treatment was significantly lower (p < 0.05) in 0.05C, 0.2C, and NS groups compared to the control immediately after 24 h of culture (control 3.76 × 10^8^, Xyl: 3.30 × 10^8^, 0.05C: 5.73 × 10^7^, 0.2C: 1.77 × 10^7^, NS: 1.87 × 10^7^). No statistically significant difference was observed in CFU between the Xyl and control groups (p > 0.05) (Fig 2a). After continuing the culture for 48 h, CFU in all groups but Xyl was statistically significantly lower than in the control (p < 0.05) (control: 4.90 × 10^8,^ Xyl: 4.67 × 10^8^, 0.05C: 1.55 × 10^8^, 0.2C: 7.10 × 10^7^, NS: 4.87 × 10^7^). Furthermore, 0.2C and NS groups showed significantly lower CFU compared to the other groups (Fig 2b).

**Table 2:** Solutions used

Solutions used	Abbreviation
Nanosilver mouthrinse	NS
0.2% Chlorhexidine gluconate	0.2C
0.05% Chlorhexidine gluconate	0.05C
25% Xylitol	Xyl


### Analysis of Lactic Acid Production

**Fig 3:**
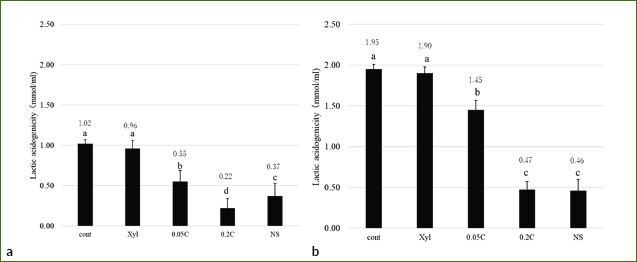
Determination of lactic acid in biofilms after treatment (A), and following 48 h cultivation after treatment (B). Values with different letters differe statistically significantly among groups (p < 0.05).

Measurements of lactate production in the biofilms showed statistically significantly less lactic acid production in all groups compared to the control and Xyl groups after 24 h of culture (p < 0.05) (control: 1.02; Xyl: 0.96; 0.05C: 0.55; 0.2C: 0.22; NS: 0.37) (Fig 3a). After continuing the culture for 48 h, an increase in lactic acid production was observed in all groups. However, the 0.2C and SV groups showed persistently lower lactate production than the other groups (p < 0.05) (control 1.95, Xyl: 1.90, 0.05C: 1.45, 0.2C: 0.47, NS: 0.46) (Fig 3b).

All data supporting the results of this study are available from the corresponding author (YM) upon reasonable request.

## DISCUSSION

The polymicrobial biofilm culture method used in this study was a modified version of the method reported by Exterkate et al.^5^ In order to simulate the initial formation of oral biofilms, an initial 24-h cultivation was performed, and the immediate effect on the biofilm was confirmed by treating it at that point; the sustained effect was examined by continuing the culture for 48 h thereafter.^20,21^ The number of viable bacterial cells following treatment was statistically significantly lower in 0.05C, 0.2C and NS groups compared to the control group (p < 0.05). However, we observed no statistically significant difference in CFU between Xyl and the control group (p > 0.05). When the culture was continued for 48 h, the number of CFU in all groups but Xyl was statistically significantly lower than in the control (p < 0.05). 0.2C and NS groups showed remarkably fewer CFU compared to all groups following 24-h culture (p < 0.05). Moreover, treatment with NS mouthrinse statistically significantly inhibited bacterial regrowth compared to treatment with 0.2% chlorhexidine gluconate after 72 h of cultivation (p < 0.05). Also, lactic acid production in the PM biofilms was statistically significantly suppressed in the 0.05C, 0.2C and NS groups compared to the control and Xyl groups following 24 h of culturing (p < 0.05), but lactic acidogenicity of NS was statistically significantly higher than that of 0.2 C (p < 0.05). Furthermore, upon continued culture for 48 h, lactic acid production increased statistically significantly across all groups (p < 0.05). On the other hand, lactic acid production in the 0.2C and NS groups remained remarkably suppressed compared to the other groups (p < 0.05). These results indicate that among the components contained in NS mouthrinse, AgNP have an antibacterial effect in biofilms equal to or greater than that of 0.2% CHX over time after treatment.

AgNP play an important role in various fields of nanoscience and nanotechnology, particularly in nanomedicine. They have diverse properties, serving as antibacterial, antifungal, antiviral, anti-inflammatory, antiangiogenic, and anticancer agents.^25^ AgNP are promising alternatives to conventional antimicrobial antibiotics due to their ability to overcome bacterial resistance. Therefore, it is necessary to develop AgNP as antibacterial agents.^25^ A mouthrinse containing AgNP can sustainably suppress bacterial reproduction and lactate metabolism.^6^ This suggests that lactate metabolism may have been suppressed in this study as well.

The antibacterial properties of silver ions derive from their unstable state, which leads to their dissociation from oxygen molecules upon contact with microorganisms. The antibacterial mechanism of silver ions is thought to be as follows: silver ions are taken into the cells of microorganisms, bind to proteins, inhibit functions such as cell division, generate active oxygen, and kill microbial cells.

Although the antibacterial mechanism of AgNP has been widely discussed, the actual facts remain unclear. AgNP are thought to interact with bacterial cells by anchoring themselves to the surface, and potentially penetrate the cell. This interaction can induce a physical change in the bacterial membrane, leading to membrane damage and leakage of cellular contents, resulting in bacterial death.^18^ Additionally, AgNP possess another antimicrobial property: they can generate high levels of reactive oxygen species and free radicals, such as hydrogen peroxide, superoxide anions, hydroxyl radicals, hypochlorous acid, and singlet oxygen.^9^ These reactive species contribute to the antibacterial activity of AgNP. Silver nanoparticles have been reported to inhibit not only the initial steps of biofilm formation, such as attachment and microcolonisation, but also the maturation steps.^11,12^ In particular, silver nanoparticles exhibit antibacterial activity against drug-resistant bacteria, with resistant strains being less likely to emerge.^1,4,19^ Therefore, even if they exert a sustained antibacterial effect, their use is likely to be biologically safe.

Furthermore, due to their property of facile cellular uptake, AgNP can be delivered close to bacteria within biofilms where conventional antibacterial agents often struggle to penetrate, as demonstrated in a recent study.^8^ Also, it was reported that antibiofilm efficacy of AgNPs was found to be statistically significant when it was used as medication, due to the prolonged interaction between positively charged AgNPs and negatively charged biofilm bacteria/structure.^24^ This suggests a potential antibacterial effect of AgNP. At antibacterial concentrations, AgNP exhibit no cytotoxicity to tested mammalian cell lines, including macrophages, stem cells, and epithelial cells. Additionally, synergistic effects with certain antibiotics have been reported.^4^

This study has some limitations. We investigated the antibacterial effect of silver nanoparticles on relatively young biofilms formed within 24 h. Since biofilms of various maturity levels exist in the oral cavity, our results do not show whether AgNP are effective against biofilms of all maturity levels. However, the PM biofilm model we used is capable of producing biofilms at various maturity levels. Thus, in the future, we would like to investigate the antibacterial effects of AgNP on biofilms at different stages of maturity.

## CONCLUSION

A mouthrinse containing silver nanoparticles suppressed bacterial viability and lactic acidogenicity in polymicrobial biofilms to an equivalent or greater degree than did 0.2% chlorhexidine gluconate over time.
